# Analysis of genetic information from the antlers of *Rangifer tarandus* (reindeer) at the rapid growth stage

**DOI:** 10.1371/journal.pone.0230168

**Published:** 2020-03-13

**Authors:** Xiaodan Bi, Jiancheng Zhai, Yanling Xia, Heping Li

**Affiliations:** 1 College of Wildlife and Protected Area, Northeast Forestry University, Xiangfang District, China; 2 College of Chemistry and Life Science, Chifeng University, Hongshan District, China; 3 School of Earth Sciences, East China University of Technology, China; Universite de Lausanne Faculte de biologie et medecine, SWITZERLAND

## Abstract

Reindeer is the only deer species in which both males and females regularly grow antlers, providing an excellent model for studying the rapid growth and annual regeneration of antlers. The study of genetic information from reindeer is the basis for revealing the unique mechanism of antler growth. In the present study, we obtained 18.86 GB of clean reads, which were assembled to obtain 94,575 unigenes (average length: 704.69). Among these reads, 30,980 sequences were identified by searching a database of known proteins and then annotated with Gene Ontology (GO) terms, Clusters of Orthologous Groups (COG) classifications and Kyoto Encyclopedia of Genes and Genomes (KEGG) pathways. All 7,480 simple sequence repeats (SSRs) were detected. A total of 84,435 and 82,226 high-quality single-nucleotide polymorphisms (SNPs) were identified in male and female reindeer, respectively. We identified 31 genes that were highly expressed in reindeer antlers. These genes regulate cell activities that are closely associated with the process of rapid tissue growth. Our results provide a basis for studying reindeer antlers and for further studying the molecular genetics, population genetics, and functional genomics of reindeer.

## Introduction

Deer antlers are appendages that undergo annual regeneration [1]. These structures are located on the fore frontal bone of animals of the Cervidae family. In general, deer antlers regenerate annually in spring, when the hard horn is cast, and then grow rapidly in summer and undergo ossification in autumn, after which the antler skin is shed, and the antlers regenerate completely in the following spring. The entire process of antler growth can be divided into the rapid growth stage and the ossification stage [2]. The rapid growth stage occurs approximately 60 days after the antler starts to grow. The extremely high rate of antler growth can reach 2 cm/day, which is one of the fastest rates of organ development in the animal kingdom [3, 4].

The growth center responsible for antler growth, located in the antler tip region [5], determines the rapid rate of antler growth and is elegantly regulated without becoming cancerous. This region was referred to be as the proliferation zone [4]. The proliferation zone consists of three layers. From distal to the proximal, these layers are the reserve mesenchyme, precartilage, and cartilage. The reserve mesenchyme is enriched with tightly packed, spindle-shaped, actively dividing cells [6, 7]. Rapid antler growth is mainly achieved through the activity of the cells residing in the proliferation zone [8]. Prince et al. found that cells in the reserve mesenchyme are in a higher undifferentiated state than those in other proximal regions [9, 10]. These cells will differentiate into chondroblasts toward the proximal region (precartilage and cartilage) [11]. Finally, bone tissues are formed through intramembranous ossification and endochondral ossification [12].

At present, little is known about the molecular mechanisms of antler regeneration and rapid growth. According to previous studies, some factors that are likely responsible for antler growth include insulin-like growth factor (IGF)-I, IGF-II and their receptors [13]; fibroblast growth factor 2 (FGF-2); transforming growth factor beta 1 (TGFβ-1); and bone morphogenetic protein (BMP)-2, BMP-4 and BMP-14 and their receptors [4, 14, 15]. IGF-I, IGF-II and the IGF receptors were first found to stimulate mesenchymal cell proliferation and were hypothesized to act as the antler growth ‘stimulus’ [13]. Members of the FGF family and their receptors have also been identified in the primary antler (first antler growth). Fibroblast growth factor 2 has been found to stimulate the proliferation of mesenchymal cells from regenerating antlers. Barling and colleagues identified BMP-2, BMP-4, BMP-14 and their receptors in the primary antler. Faucheux et al. performed immunolocalization of TGF-β in regenerating antlers. Some studies have shown that BMP-2 and TGFβ-1 inhibit the proliferation of mesenchymal cells, but other functionally significant components require further study. A previous study presented several lines of evidence showing that retinoic acid (RA) also plays a role in controlling mesenchymal cell growth and differentiation; sequence information related to antler growth needs to be further examined.

Reindeer are a unique cervid species in which both males and females annually regenerate antlers from the permanent cranial bony protuberance (pedicle). In reindeer antlers, the histologically classified zones are similar to those of other major deer species, including white-tailed deer, mule deer, fallow deer, red deer, American elk, caribou, and sika deer [11]. However, the reindeer antler growth process is poorly understood, and there has been no correlational research on the molecular mechanism underlying the rapid growth of reindeer antlers. Sequencing the transcriptomes of tissues is an effective way to generate useful sequence information to further reveal the molecular mechanisms underlying antler growth and development. In this study, we used RNA-Seq technology to obtain genetic information on reindeer antlers in the rapid growth stage. This investigation on the antlers of reindeer will greatly enhance our understanding of the molecular mechanisms underlying antler growth.

## Materials and methods

### Ethics statement

All procedures used in this study were approved by the Institutional Animal Care and Use Committee of Northeast Forestry University (Harbin, China) (UT-31; 90 20 June 2014).

### Sample collection and preparation

**Fig 1:**
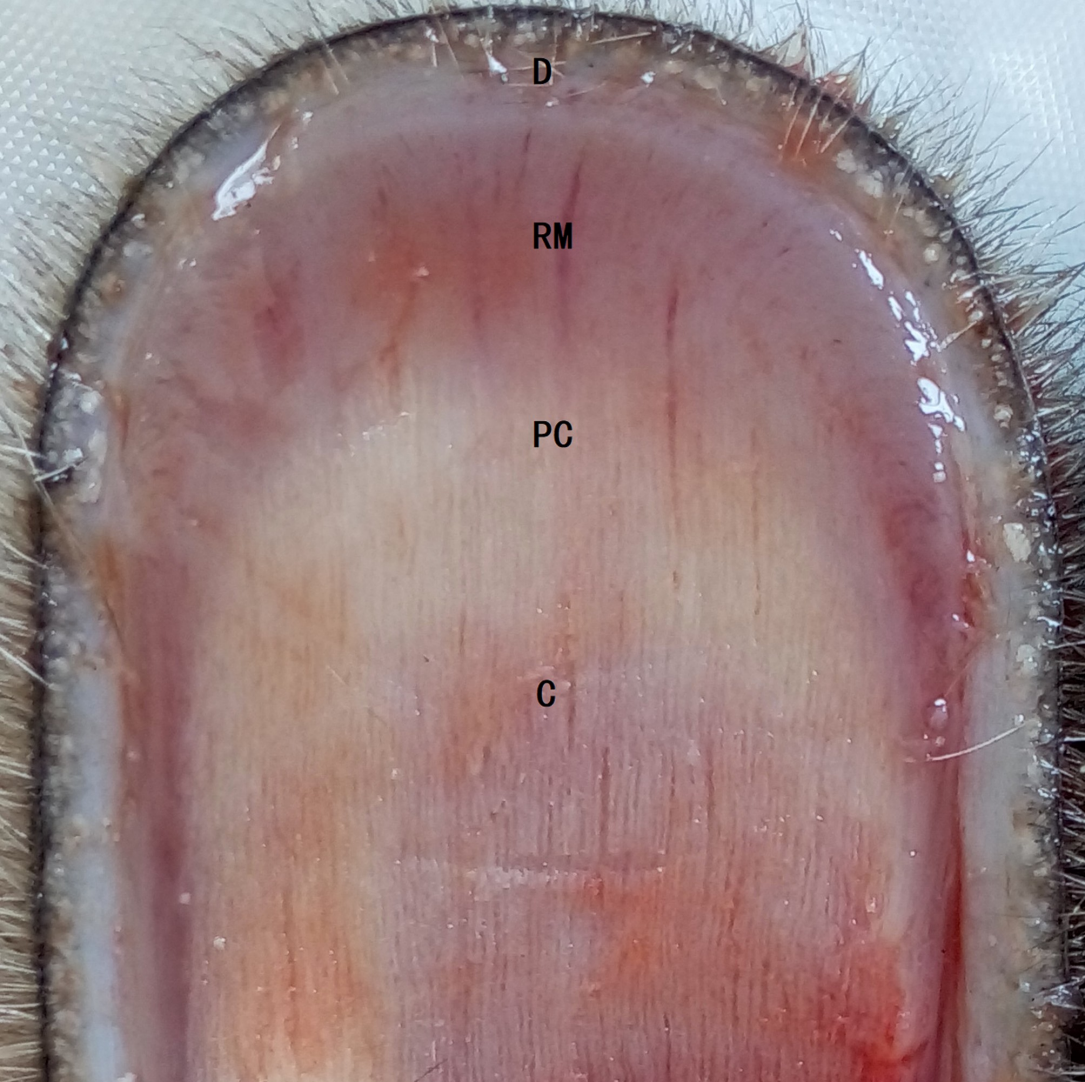
Identification of the proliferative zone in a growing reindeer antler tip. D, dermis; RM, reserve mesenchyme; PC, precartilage; C, cartilage.

Antler tips were collected from three anesthetized 6-year-old female and male reindeer (over 60 days) at the Liaoyang deer farm in Liaoning Province. These reindeer were introduced from the Ewenki reindeer herd of Aoluguya, Inner Mongolia, China. The antler mesenchyme layer (Fig 1) was cut into small pieces according to a previously reported method [8], and the samples were immediately placed in RNA storage solution. Subsequently, the samples were stored at -80°C in the laboratory until further use.

### Total RNA preparation

Three samples of antler mesenchyme were pooled into one sample for extraction of total RNA using Trizol reagent (Invitrogen) according to the manufacturer’s protocol. RNA concentration was measured using the Qubit^TM^ RNA Assay Kit in a Qubit^®^2.0 fluorometer (Life Technologies, CA, USA). RNA integrity was assessed using the RNA Nano 6000 Assay Kit and an Agilent Bioanalyzer 2100 system (Agilent Technologies, CA, USA).

### Library preparation and Illumina sequencing

Sequencing libraries were generated using the NEBNext^®^Ultra^™^ RNA Library Prep Kit for Illumina^®^ (NEB, USA) following the manufacturer’s recommendations. First, mRNA was purified from total RNA using oligo(dT) magnetic beads and fragmented into small pieces in NEBNext First Strand Synthesis Reaction Buffer (5X). First-strand cDNA was synthesized using random hexamer primers and M-MuLV reverse transcriptase (RNase H-). Second-strand cDNA synthesis was subsequently performed using DNA polymerase I and RNase H. NEBNext Adaptor with hairpin loop structures were ligated to prepare for hybridization. The library fragments were purified with the AMPure XP system (Beckman Coulter, Beverly, USA). PCR was then performed with Phusion high-Fidelity DNA polymerase, universal PCR primers and the Index (X) primer. Finally, the PCR products were purified (AMPure XP system), and library quality was assessed in an Agilent Bioanalyzer 2100 system. The library preparations were sequenced on the Illumina HiSeq platform, and paired-end reads were generated.

### Data quality control

In this step, clean data (clean reads) were obtained by removing reads containing adapters, reads containing poly-N sequences and low-quality reads from the raw data. All downstream analyses were based on clean data with high quality.

### Transcriptome assembly

Clean short reads were used for the de novo assembly of reindeer unigenes. De novo assembly was conducted using Trinity software. The longest transcript from the potential alternative splicing transcripts was selected as the sample unigene. The unigenes of two samples were combined to create a unigene database.

### Functional annotation of unigenes

The general unigene sequences were subjected to BLAST searches against the NCBI nonredundant (Nr) protein database and the SwissProt, Gene Ontology (GO), Clusters of Orthologous Groups (COG), Eukaryotic Orthologous Groups (KOG), EggNOG, and Kyoto Encyclopedia of Genes and Genomes (KEGG) databases. Using KOBAS2.0 software, annotation of unigenes according to KEGG Orthology was performed. After amino sequence prediction, annotation of unigenes was performed using HMMER software against the Pfam database.

### Structural annotation of unigenes

#### a) SSR identification

Unigenes ≧1 kb were subjected to simple sequence repeat (SSR) analysis with the microsatellite identification tool MISA (http://pgrc.ipk-gatersleben.de/misa/). The detection criteria were constrained to perfect repeat motifs of 1–6 bp for mono-, di-, tri-, tetra-, penta- and hexa-nucleotide microsatellites, respectively.

#### b) SNP calling

Picard Tools v1.41 and SAMtools v0.1.18 were used for sorting, removal of duplicated reads and merging of the BAM alignment results of each sample. GATK2 software was used to perform single-nucleotide polymorphism (SNP) calling. Raw vcffiles were filtered with the GATK standard filter method and the following parameter settings: clusterWindowSize: 35; MQ0 ≥4 and (MQ0/ (1.0*DP)) > 0.1; QUAL < 10; QUAL < 30.0, QD < 5.0 or Hrun > 5. Only SNPs with a distance > 5 were retained.

### Calculation of gene expression levels

The reads obtained from sequencing were compared with the unigene library using Bowtie [16], and expression levels were estimated using RSEM [17] according to the comparison results. Gene expression levels were expressed as FPKM (fragments per kilobase of transcript per million mapped reads) values using the formula FPKM = 10^9^C/NL. In this equation, C is the number of mappable fragments that were uniquely aligned to a unigene; N is the total number of mappable fragments that were uniquely aligned to all unigenes; and L is the length of a unigene in base pairs.

### Quantitative real-time PCR analysis

Real-time PCR (qPCR) assays were performed by using the One-Step SYBR method in a 7500 Real-Time PCR system to test the gene expression levels. The expression levels of target genes were normalized against the internal reference gene β-actin. Relative gene expression was calculated using the 2^–ΔΔCt^ method [18].

## Results

### Illumina sequencing, assembly, and sequence analysis

cDNA samples were extracted from a male reindeer antler (SRR9051566) and a female reindeer antler (SRR9051567). Using the Illumina sequencing platform, a number of raw reads were generated from the two samples (S1 Table). Clean reads were obtained by removing low-quality reads and adaptor sequences. The amount of total clean data for the assembly of unigenes was 18.86 GB. These clean data were assembled to obtain 124,139 transcripts and 94,575 unigenes. The mean lengths of the transcripts and unigenes were 911.37 nt and 704.69 nt, respectively (Table 1).

**Table 1:** Statistics of unigene assembly.

Length Range	Transcripts	Unigenes
200–300	38,259 (30.82%)	35,811 (37.87%)
300–500	30,083 (24.23%)	25,666 (27.14%)
500–1000	25,025 (20.16%)	17,505 (18.51%)
1000–2000	16,323 (13.15%)	8,712 (9.21%)
2000+	14,449 (11.64%)	6,881 (7.28%)
Total Number	124,139	94,575
Total Length	113,136,185	66,645,778
N50 Length	1,768	1,193
Mean Length	911.37	704.69

### Annotation of predicted proteins

**Fig 2:**
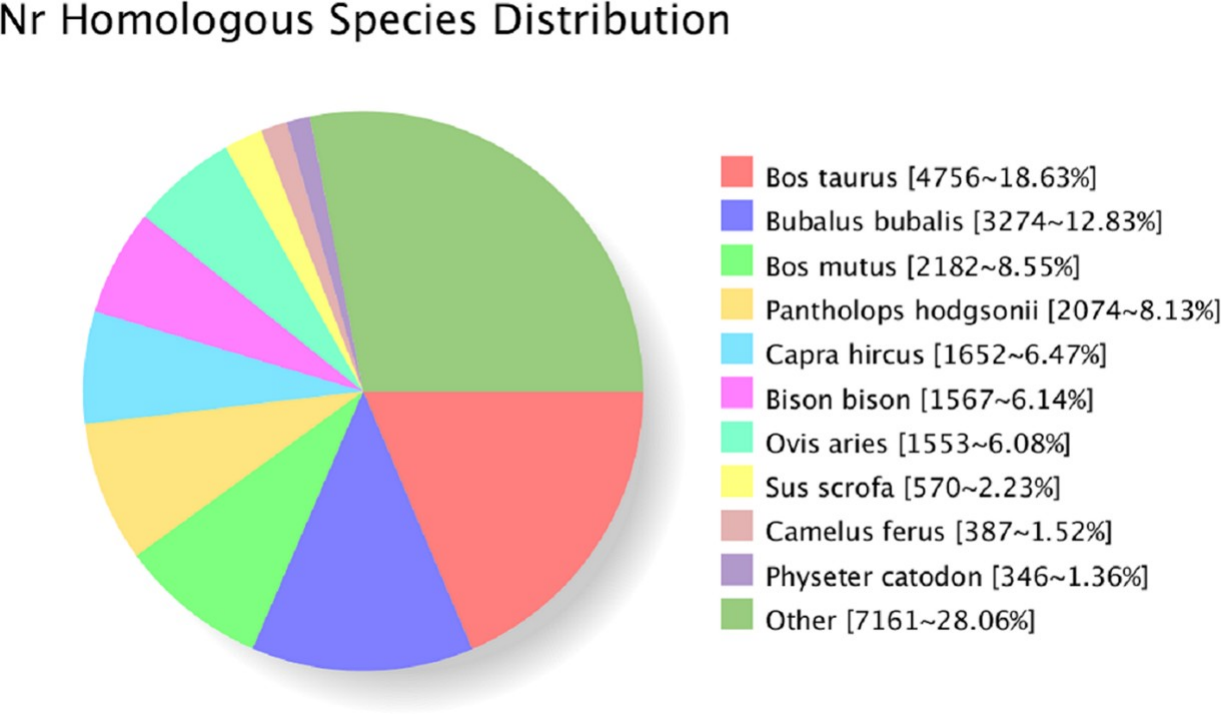
Species distribution of unigene BLAST results. Note: this figure shows the species distribution of unigene BLASTx results against the NCBI-Nr protein database with a cutoff E value of 10–5. Different colors represent different species.

These sequences were first searched using BLASTx against the NCBI nonredundant (Nr), SwissProt, GO, COG, KOG, EggNOG, and KEGG protein databases with a cutoff E value of 10–5. A total of 30,980 unigene annotations were obtained (Table 2). The Nr database queries revealed that the highest percentages of matched sequences were 18.63% for *Bos taurus*, 12.83% for *Bubalus bubalis*, 8.55% for *Bos mutus*, 8.13% for *Pantholops hodgsonii*, 6.47% for *Capra hircus*, 6.14% for *Bison bison* and 6.08% for *Ovis aries*. The percentages of sequences matched to other species did not total 35% (Fig 2).

**Table 2:** Annotation of unigenes.

Anno Database	Annotated Number	300≤Length<1000	Length≥1000
COG Annotation	7,648	2,732	4,093
GO Annotation	14,618	4,949	8,298
KEGG Annotation	15,167	5,820	7,608
KOG Annotation	18,602	7,268	8,817
Pfam Annotation	18,928	6,931	10,138
SwissProt Annotation	15,612	5,535	8,650
EggNOG Annotation	27,389	11,407	11,697
Nr Annotation	25,555	10,320	11,813
All Annotated	30,980	13,491	12,156

### Gene functional annotation

Based on the GO classifications, 14,618 sequences were categorized into 60 functional groups. In the three main categories of the GO classification, namely, biological process, cellular component and molecular function, the predominant terms were “cell part (10,368 members)”, “cellular process (9,375 members)”, and “binding (8,839 members)”, respectively. The terms “single-organism process (8,454 members)”, “organelle (7,900 members)”, “catalytic activity (5,158 members)” and “metabolic process (7,174 members)” were associated with the highest percentages of genes. Few genes were found in the clusters of “morphogen activity (1 member)”, “protein tag (1 member)”, “virion part (2 members)”, “chemorepellent activity (3 members)” and “metallochaperone activity (5 members)” (S1 Fig).

We also classified the 7,648 annotated sequences into 25 COG categories. The cluster for “general function prediction only (2,509 members)” was the largest group, followed by “transcription (938 members)”, “replication, recombination and repair (909 members)”, and “translation, ribosomal structure and biogenesis (721 members)”. The categories representing extracellular structures did not contain any members, and nuclear structure (4 members) was the smallest group (S2 Fig). We also annotated 30,980 sequences to the reference pathways in the KEGG database. There were 15,167 sequences mapped to 292 KEGG pathways (S2 Table).

### Analysis and statistics of SSRs

Using MISA software, SSR analysis of unigenes with lengths greater than 1 kb was performed, and the results are shown in Table 3. We identified 7,480 SSRs distributed in 5,955 sequences, accounting for 5.91% of the total number of unigenes. Mononucleotide repeats were the most common microsatellite form present (58.70%), followed by trinucleotide repeats (19.30%) and dinucleotide repeats (14.30%). Among the mononucleotide repeats, the (A)n and (G)n types were the most common repeats. The (AC)n motif was the most frequently recovered dinucleotide repeat. The numbers of GGC/GCC/GCG/ CCG/CGC/ CGG-type repeats were greater than those of other trinucleotide repeats, and these motifs are rich in GC base repeats. The percentages of other types of repeats (compound formation, tetranucleotide, pentanucleotide, hexanucleotide) among all SSRs did not reach 1%.

**Table 3:** Summary of simple sequence repeat (SSR) types in the reindeer transcriptome.

SSR type	Repeat motif	Number	All/frequency
C		491	491/6.40%
C*		7	7/0.09%
P1	A/T /G /C	2019/2008/202/160	4389/58.70%
P2	AC/AT/TG/CA/GT/TA/TC/AG/GA/CT/GC/CG/	152/146/144/143/127/103/62/53/52/39/29/20	1070/14.30%
P3	GGC/GCC/GCG/ CCG/CGC/ CGG/ GAG/ CAG/GCT/GCA/CTG/GGA/TCC/AGC/CCT/AGG/TGC/CTC/CTT/TCT/TTG/AAT/TTC/ CCA /AAG/ GGT/GTG/CAC/GAA/AAC/TCA/TGA/ AGA/TGG/ GAT/ATC/ATT/TTA/ACC/CAA/TAA/TAT/ATA/ATG/CAT/ GTT/TGT/ GAC/ACA/CGT/ACG/ /CGA/TAG/TCG	164/138/116/88/83/83/60/55/55/47/46/40/40/38/37/32/30/29/17/14/14/14/13/12/12/12/12/11/10/9/9/9/9/8/8/7/6/6/5/5/5/5/4/4/4/3/3/2/2/2/1/1/1/1	1446/19.30%
P4	TGAA/TTTC/TTTG/AAAC/AATA/AGAC/ CAGA/GGAC/TTTA/ /GGGA/AACA/AACC/ AAGG/ACAA/AGCC/AGGA/AGGC/ ATAA/ATCC/ATGG/ATTT/CAAA/ CTGC /CTGG/GAAT/GACA/GATG/GCAG/GCCA/GCCT/GCTC/GGAC/ GAG/GGCA/ GGCG/GGCT/GGGC/GTGA/GTGC/GTTG /GTTT/TAAA/TATG/TATT/TCTG/TCGC/TGAC/TGAG/TGTA/TGTC/TGTT/TTCA/TTCC/TTGG	3/3/3/2/2/2/2/2/2/2/1/1/1/1/1/1/1/1/1/1/1/1/1/1/1/1/1/1/1/1/1/1/1/1/1/1/1/1/1/1/1/1/1/1/1/1/1/1/1/1/1/1/1/1	68/0.90%
P5	AAACT/AGGGG/CCGCC/GCGCC/GGGCC/TGCCC	1/1/1/1/1/1	6/0.08%
P6	AGGACG	1	1/0.01%
Total		7,480	

Note: C and C*: compound SSRs, P1: mononucleotide SSR, P2: dinucleotide SSR, P3: trinucleotide SSR, P4: tetranucleotide SSR, P5: pentanucleotide SSR, P6: hexanucleotide SSR; Total, total number of SSRs.

### SNP calling analysis

The sequencing reads were named according to the SNP locus allele numbers (Allele). SNP loci can be divided into homozygous-type SNP loci (only one allele) and heterozygous-type SNP loci (two or more alleles) (Table 4). The GATK2 calls generated a total of 84,435 and 82,226 high-quality SNPs for the male and female reindeer, respectively. All 50,100 SNPs were shared by the male and female reindeer and were classified into 3 types: 4,689 homozygous-type SNP loci in both males and females, 25,110 that were homozygous in males and heterozygous in females, and 20,301 that were heterozygous in males and homozygous in females.

**Table 4:** Quantitative SNP statistics.

Samples	HomoSNP	HeteSNP	AllSNP
Male	26,818	57,617	84,435
Female	31,272	50,954	82,226

Note: HomoSNP: homozygous-type SNPs; HeteSNP: heterozygous-type SNPs; AllSNP: homozygous-and heterozygous-type SNPs.

### Detection of gene sequences involved in rapid antler growth

By calculating gene expression levels, we characterized a large number of genes that were highly expressed during the rapid growth stage. The results of the real-time PCR (qPCR) assays were consistent with the transcriptome sequencing results (S3 Fig). These highly expressed genes were related to rapid tissue growth and included genes encoding signaling molecules, transcription factors and extracellular matrix (ECM) components (Tables 5–7).

**Table 5:** Summary of genes with signal transduction functions involved in rapid growth.

Gene name	RPKM	
	Male	Female
Cyclin-dependent kinase inhibitor 1B (*CDKN1B*)	3004.986	2536.775
Cyclin-dependent kinase inhibitor 1C (*CDKN1C*)	489.1891	167.1021
Pleiotrophin (*PTN*)	383.7536	778.8729
Gap junction protein, alpha 1, 43 kDa (*GJA1*)	382.4876	486.3553
Mortality factor 4-like protein 2 (*MORF4L2*)	191.064	221.8099
Indian hedgehog protein (*IHH*)	200.3888	66.47308
Midkine (*MDK*)	111.7767	235.8324
Endoglin (*ENG*)	96.55723	105.1112
Transforming growth factor beta 1 (*TGFB1*)	28.85541	22.0079

**Table 6:** Summary of genes with transcription factor functions involved in rapid growth.

Gene name	RPKM	
	Male	Female
Cyclic AMP-dependent transcription factor ATF-4 (*ATF4*)	174.4363	217.905
Transcription factor AP-1 (*AP-1*)	64.64342	102.5571
Runt-related transcription factor 2 (*RUNX2*)	83.96631	98.85752
Homeobox protein DLX-5 (*DLX5*)	62.46803	62.99823
Transcription factor SOX-9 (*SOX-9*)	24.72796	17.72542

**Table 7:** Summary of genes encoding extracellular matrix (ECM) components involved in rapid growth.

Gene name	RPKM	
	Male	Female
Tenascin-N (*TNN*)	2797.14	1421.071
Collagen alpha-1(V) chain (*COL5A1*)	1445.031	1075.73
Galectin-1 (*LGALS1*)	1386.429	1491.172
Collagen alpha-1(II) chain (*COL2A1*)	1096.858	78.31919
Collagen alpha-1(VI) chain (*COL6A1*)	1008.863	1083.736
Collagen alpha-3(VI) chain (*COL6A3*)	750.9724	922.2885
Collagen alpha-2(V) chain (*COL5A2*)	618.4498	482.3582
Bone sialoprotein II (*IBSP*)	300.7032	101.6669
Collagen alpha-1(IV) chain (*COL4A1*)	228.0083	237.0309
Collagen alpha-2(IX) chain (*COL9A2*)	117.0704	11.9906
Collagen alpha-1(XVIII) chain (*COL18A1*)	72.6827	97.51243
Collagen alpha-1(XV) chain (*COL15A1*)	68.00377	50.56384
WNT1-inducible-signaling pathway protein 1 (*WISP1*)	47.55491	38.82326
Collagen alpha-1(XXVII) chain (*COL27A1*)	36.42511	27.65785
Collagen alpha-1(III) chain (*COL3A1*)	25.04221	30.17576
Collagen alpha-1(XIII) chain (*COL13A1*)	20.18198	12.05025
Osteopontin (*OPN*)	15.52438	17.39749

## Discussion

Reindeer are the only species in which females regularly grow antlers. The Aoluguya reindeer represents one of the southernmost reindeer species in the world and the only reindeer population in China; this species is limited to a small region in the northeastern part of the Greater Khingan Mountains and numbers less than 1000 individuals [19]. Such small groups are threatened with extinction. It is necessary to carry out research on reindeer in China to protect and utilize reindeer populations. However, little is known about the underlying genetic basis of some traits of reindeer. In 2017, the reindeer genome size was published (2.64 G), and the clustering results showed that reindeer, domestic cattle and sheep share a common ancestor [20]. Our results show that the gene sequences of reindeer share the highest homology with those of *B*. *taurus*. There are a large number of SSRs and SNPs distributed in reindeer genes, and the numbers of SNPs distributed in male and female reindeer genes are different. These SSRs and SNPs are expected to serve as molecular markers for studying the genetic diversity and genetic structure of reindeer populations, which will provide guidelines for the conservation of reindeer populations. Our sequence annotation showed that 14,618 sequences were categorized into 60 GO classifications; 7,648 sequences were categorized into 25 COG classifications; and 15,167 sequences were categorized into 292 KEGG pathways. There are 4 pathways related to cellular activities, including pathways associated with cancer (88 members), the PI3K-Akt signaling pathway (427 numbers), focal adhesion (338 members) and the MAPK signaling pathway (322 members). The annotation information provides a reference for further study of the genes that play a role in the rapid growth of reindeer antlers.

Recently, comparative genomic studies have shown that there are gene mutants that are specifically expressed in the reindeer genome [21]. Research shows that tumor suppressor genes and proto-oncogenes are strongly positive for selection in cervids and exhibit strong specific expression in the antlers (e.g., *ADAMTS18*, *FOS*, *REL*, and *FAM83A*), also find that the fast-growing antlers present a more osteosarcoma-like profile than normal bone tissue [22]. In this study, we observed that a number of genes were highly expressed in the reserve mesenchyme of reindeer antlers, including nine genes encoding signaling proteins (Table 5). Five of these signaling proteins (CDKN1B, CDKN1C, PTN, GJA1, and MORF4L2) can act as tumor suppressors. The CDKN1B gene exhibited the highest expression level. The major functions of CDKN1B and CDKN1C are to stop or slow down the cell division cycle and control cell cycle progression at G1 [23]. The PTN gene is highly expressed in several tumor cell types [24] involved in tumor angiogenesis and presents mitogenic activity for fibroblasts [25]. GJA1 can regulate cell death, proliferation, and differentiation [26]. MORF4L2 may be required for replicative senescence, apoptosis, and DNA repair. The other four genes (IHH, MK or MDK, ENG, and TGF-βs) perform many cellular functions, including controlling cell growth, cell proliferation, cell differentiation, apoptosis, migration and adhesion [27–29].

Five transcription factor genes (ATF4, AP-1, RUNX2, DLX-5, and SOX-9) were identified (Table 6). ATF4 was the most highly expressed transcription factor, similar to the findings of a study on the antler tips of Chinese sika deer [30]. Wang W et al. found that ATF4 is expressed in growth plate chondrocytes and controls chondrocyte proliferation and differentiation. AP-1 has been shown to control cellular processes, including differentiation, proliferation, and apoptosis [31–33]. RUNX2 plays a cell proliferation-related regulatory role in cell cycle entry and exit in osteoblasts. This protein suppresses preosteoblast proliferation by affecting cell cycle progression in the G1 phase and inhibits osteoblastic differentiation via DNA binding [34]. DLX-5 is necessary for osteoblast differentiation as an early BMP-responsive transcriptional activator [35]. The transcription factor SOX-9 is crucial for chondrocyte differentiation [36].

We also observed seventeen ECM protein genes that were highly expressed (Table 7), mainly including TN-N, COLA, Gal-1, WISP-1, OPN, and BSPII. TN-N was the most highly expressed ECM protein gene. Previous research has shown that TN-N is able to induce the migration of tumor cells as well as endothelial cells [37]. Gal-1 is a lectin with a broad range of biological activities [38]. Recently, a study suggested that total absence of Gal-1 in the bone microenvironment allows rapid development of bone tumors [39]. Increased expression of Gal-1 has been correlated with a variety of processes in cancer progression, including cellular aggregation, tumor formation, the metastatic spread of cancer, angiogenesis, and apoptosis [40]. OPN and BSPII belong to a family of glycoproteins that have been linked to cancer metastasis and progression and are overexpressed in a variety of cancers, including lung cancer, breast cancer, colorectal cancer, stomach cancer, ovarian cancer, papillary thyroid carcinoma, melanoma and pleural mesothelioma [41, 42]. WISP-1 promotes mesenchymal cell proliferation and osteoblastic differentiation and represses chondrocytic differentiation [43, 44]. Among these ECM protein genes, 10 types of *COLA* genes were highly expressed, namely, type II, III, IV, V, Ⅵ, Ⅸ, XIII, XV, XVIII, and XXVII genes. In our laboratory, using the differential display reverse transcription PCR method, Zhai Jiancheng et al. previously found that COL6A3 exhibits a high expression level in the mesenchymal layer of male and female reindeer antlers [45]. In the present study, COL6A3 was found to be widely expressed in both male and female antlers. Notably, collagen II, which is a marker of mature cartilage, was shown to be highly expressed in the reindeer antler during the period of rapid antler growth (60 days) in the present study. Rucklidge GJ et al. previously demonstrated that collagen type II is expressed in young antlers (6 weeks) in small quantities and is not expressed in old antlers (5 months) [46]. The collagen II expression pattern in reindeer antlers needs to be explored in further detail. The composition and function of the ECM play an important role in anticancer effects in naked mole rats [47]. The antler mesenchymal layer expresses a large number of ECM genes, the functions of which need to be further studied.

## Conclusions

Our results are identical to those of a previous study showing that the gene sequences of reindeer share higher homology with those of *B*. *taurus* than with those of other species. The number and distribution of SNPs are different in male and female reindeer. In the mesenchymal layer of the antler tip in male and female reindeer, a large number of highly expressed genes may be related to rapid antler growth. Perhaps these genes are commonly involved in activities such as cell proliferation and differentiation control. Some tumor suppressors are highly expressed during rapid antler growth. The study of the correlation between rapid antler growth and tumor progression may provide a new perspective on the mechanism of antler development.

## Supporting information

S1 Fig GO categories of the unigenes. The results are summarized in three main categories: biological process, cellular component a molecular function. The righty-axis indicates the number of genes in a category. The lefty-axis indicates the percentage of a specific category of genes in that main category.
(TIF)

S2 Fig COG categories of the unigenes. The histogram shows the distribution of sequences among different COG categories: 7,648 sequences have a COG classification among the 25 categories.
(TIF)

S3 Fig Validation of gene expressed level by Real-time PCR. The x-axis indicates genes names. The y-axis indicates the fold changes of the genes expression. Y axis in the left (blue bars) presented the fold change of transcritome sequencing and y axis in the right (orange bars) presented the fold change of Real-time PCR.
(TIF)

S1 Table Summary for raw reads of two samples. (XLSX)

S2 Table KEGG annotation of unigenes. (XLSX)
