# Supplementary figures and images for: Analysis of genetic information from the antlers of Rangifer tarandus (reindeer) at the rapid growth stage
Source: PLoS One. 2020 Mar 13;15(3):e0230168. doi: 10.1371/journal.pone.0230168 (PMC7069613; doi:10.1371/journal.pone.0230168)

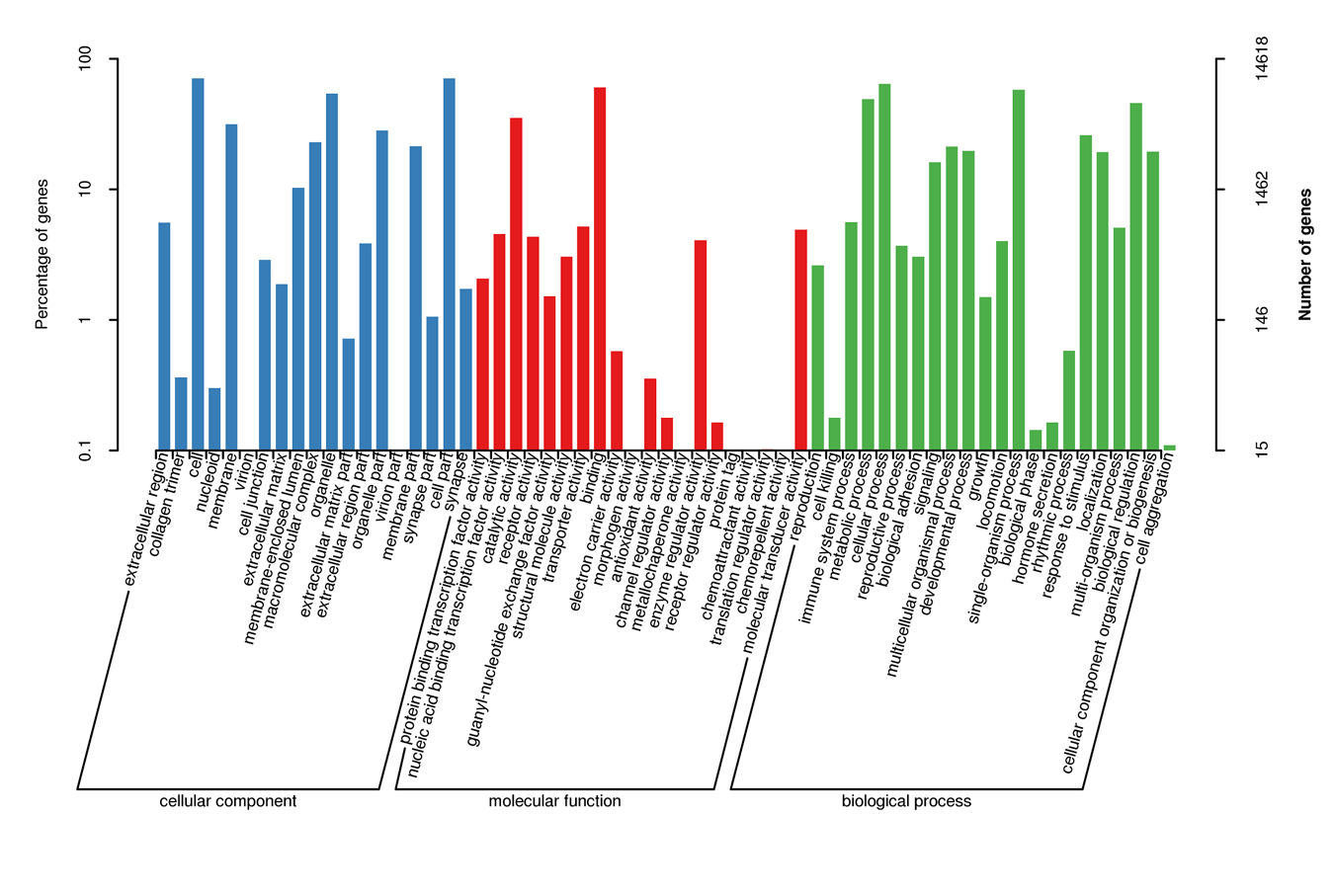

Supplement: S1 Fig — The results are summarized in three main categories: biological process, cellular component a molecular function. The righty-axis indicates the number of genes in a category. The lefty-axis indicates the percentage of a specific category of genes in that main category. (TIF) [file pone.0230168.s001.tif]

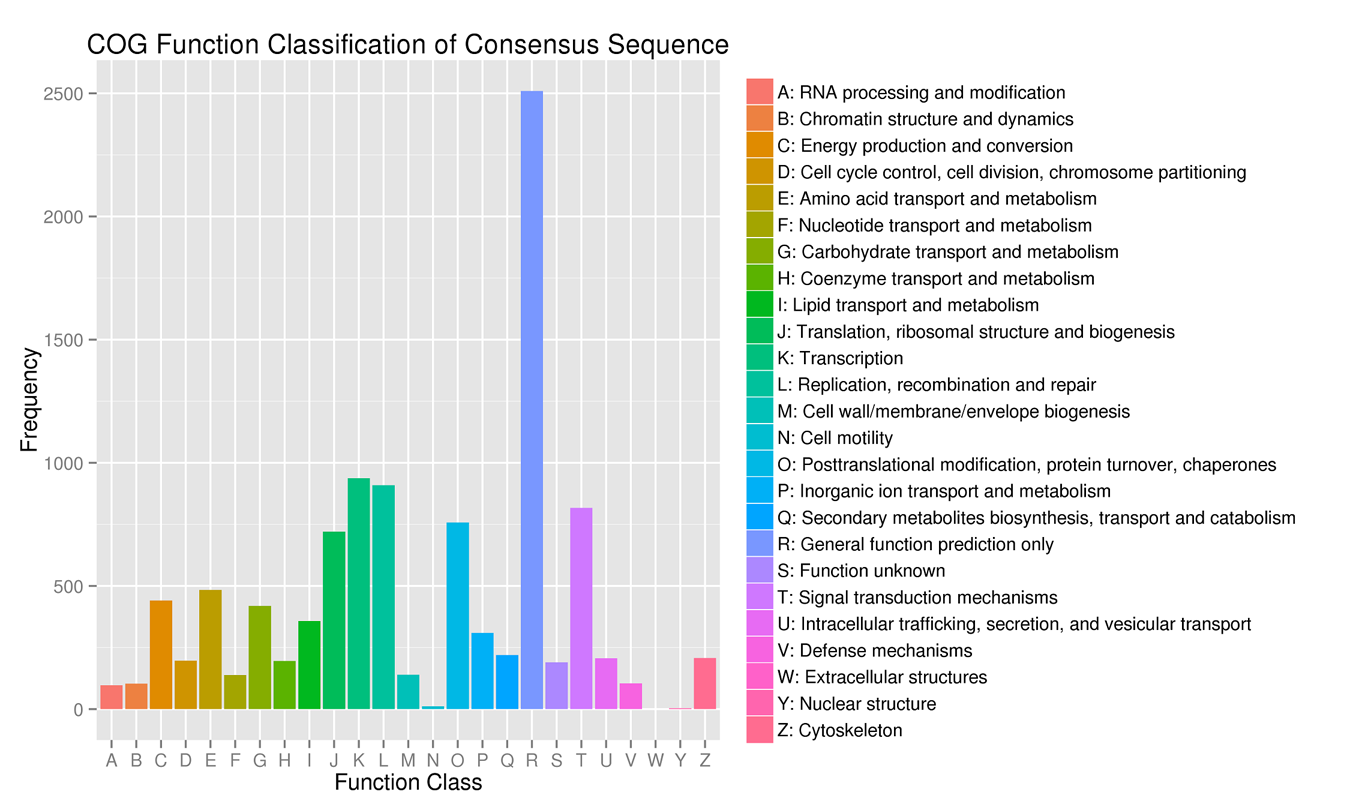

Supplement: S2 Fig — The histogram shows the distribution of sequences among different COG categories: 7,648 sequences have a COG classification among the 25 categories. (TIF) [file pone.0230168.s002.tif]

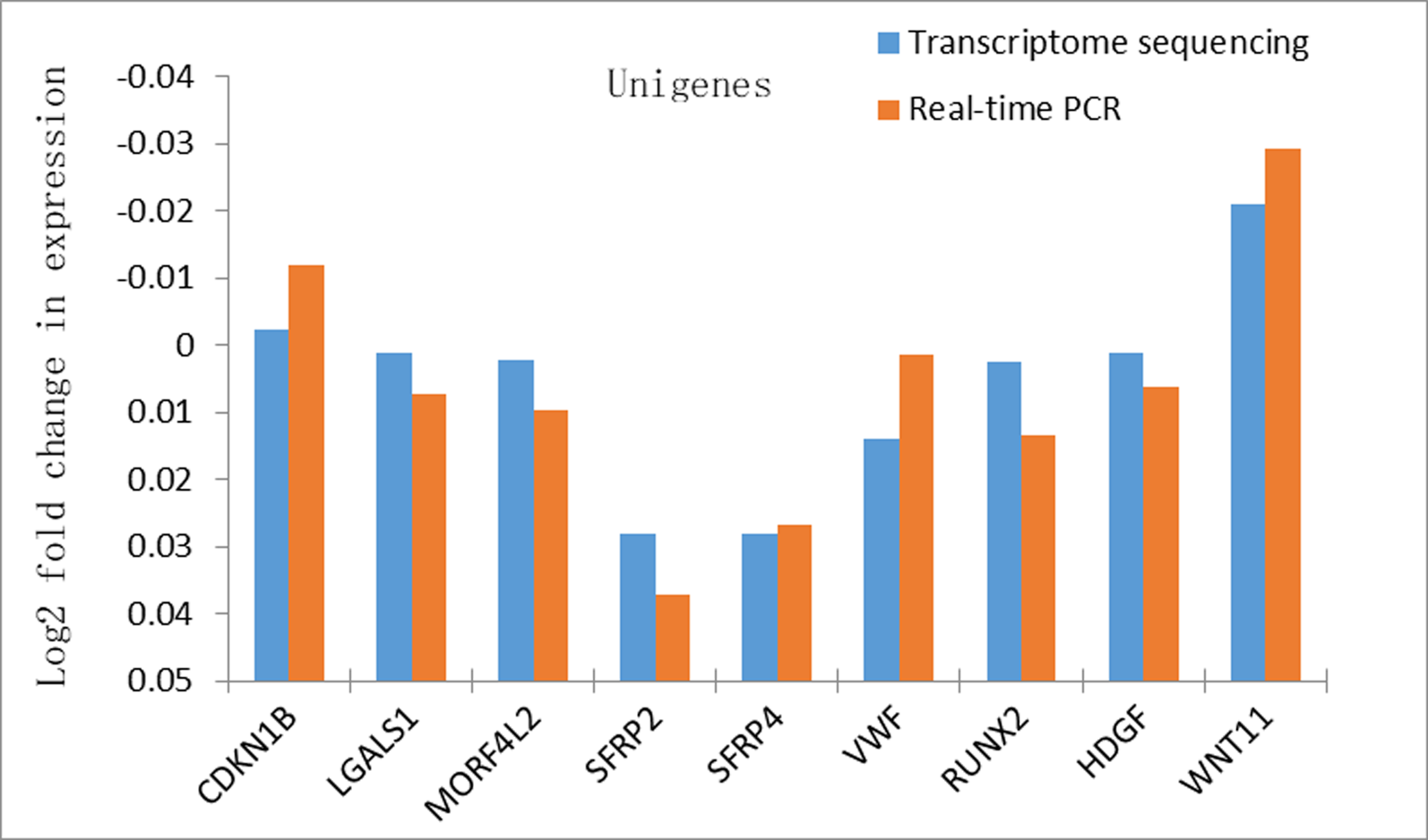

Supplement: S3 Fig — The x-axis indicates genes names. The y-axis indicates the fold changes of the genes expression. Y axis in the left (blue bars) presented the fold change of transcritome sequencing and y axis in the right (orange bars) presented the fold change of Real-time PCR. (TIF) [file pone.0230168.s003.tif]
